# Untargeted Sweat and Sebum Volatilomics by HS-SPME-GC/ToF-MS for the Identification of SARS-CoV-2-Associated Biomarkers

**DOI:** 10.3390/metabo16030158

**Published:** 2026-02-27

**Authors:** Edoardo Longo, Emanuele Boselli, Giovanni Baldassarre, Emanuela Sozio, Lucrezia Zuccarelli, Carlo Tascini, Bruno Grassi, Stefano Cesco

**Affiliations:** 1 Faculty of Agricultural, Environmental and Food Sciences, Free University of Bolzano, 39100 Bolzano, Italy; edoardo.longo@unibz.it (E.L.); stefano.cesco@unibz.it (S.C.); 2 Department of Medicine, University of Udine, 33100 Udine, Italy; baldassarre.giovanni@spes.uniud.it (G.B.); lucrezia.zuccarelli@uniud.it (L.Z.); carlo.tascini@uniud.it (C.T.); bruno.grassi@uniud.it (B.G.); 3 Division of Infectious Diseases, Azienda Sanitaria Universitaria Friuli Centrale, 33100 Udine, Italy; emanuela.sozio@asufc.sanita.fvg.it

**Keywords:** non-invasive biomarkers, volatilomics, SARS-CoV-2, HS-SPME-GC-MS, chemometrics, sweat, sebum, human metabolomics, precision medicine

## Abstract

**Background/Objectives**: The COVID-19 pandemic has emphasized the urgent need for non-invasive diagnostic strategies. While breath analysis has been widely investigated, sweat and sebum remain largely unexplored, despite being abundant, chemically diverse, and easily collected. This exploratory study presents a proof-of-concept workflow to evaluate their potential for infection biomarker discovery. **Methods**: Samples from 51 subjects were analyzed by headspace solid-phase microextraction coupled with gas chromatography and time-of-flight mass spectrometry (HS-SPME-GC/ToF-MS). Over 8000 untargeted volatile compounds were detected, reflecting the high complexity of these matrices. **Results**: Data refinement and chemometric modelling using principal component analysis (PCA) and partial least squares discriminant analysis (PLS-DA) revealed robust separation between SARS-CoV-2-positive Patients and Controls. Classification accuracies consistently exceeded 95%, demonstrating the robust discriminative performance of the approach. Among the detected volatiles, 2-methylbenzenemethanol acetate emerged as the most informative compound, representing a potential biomarker candidate. **Conclusions**: This work shows that the sweat and sebum volatilome can be exploited for clinical applications. The workflow integrates non-invasive sampling, comprehensive chromatographic profiling, and advanced statistical modelling, representing a methodological contribution to analytical chemistry. Beyond COVID-19, the strategy provides a potential framework for volatile organic compound (VOC)-based diagnostics across different diseases and supports future development of sensor technologies for translation into healthcare practice.

## 1. Introduction

The human body emits hundreds of volatile organic compounds (VOCs) through biological fluids, such as breath, saliva, feces, urine, sebum, and sweat [1,2]. These VOCs are referred to as the volatilome and offer a unique chemical fingerprint reflecting the metabolic activity and conditions of the body, contributing to a person’s specific odor [3]. The composition of the volatilome is dynamic and closely related to health conditions, diet and lifestyle, metabolism, microbiome activity, and overall physiological states. Because it reflects complex metabolic and microbial interactions, the volatilome composition can be highly informative because it may provide valuable insights into an individual’s physiological state. Changes in volatile composition can indicate shifts in metabolic processes and may be associated with various health conditions. For example, changes in the VOCs profile have been reported in cases of infections [4,5,6,7], or complex pathological conditions, such as Parkinson’s disease [8,9] and neoplastic disorders [10]. The presence or absence of specific VOCs can thus be used to detect alterations in metabolic states, making them useful indicators for disease detection and monitoring.

High-resolution techniques such as headspace solid-phase microextraction coupled with gas chromatography and mass spectrometry (HS-SPME-GC-MS) have proven particularly effective in VOCs analysis due to their high analytical sensitivity, reproducibility, and compatibility with multivariate statistics [11]. HS-SPME-GC-MS has been widely adopted across diverse fields, from environmental monitoring to the food and beverage industry, where it is used to detect trace compounds with remarkable accuracy [12], and it is now being increasingly applied in biomedical contexts. Its successful application in these areas has supported the extension of its use in clinical diagnostics, enabling the comprehensive characterization of a subject’s or patient’s volatilome [2,8,9] using an almost non-invasive procedure. As further evidence of this diagnostic value, even trained dogs have demonstrated the ability to detect diseases by recognizing specific VOCs patterns in body fluids, further highlighting the diagnostic relevance of volatilome characterization [5]. The non-invasive nature of VOCs sampling, combined with its potential for high specificity and sensitivity, represents a notable advantage, particularly for populations where traditional diagnostic methods may be impractical. For example, bacterial infections in the lungs have been detected in vivo by sampling the breath and analyzing the emitted VOCs, allowing the specific diagnosis of infections from closely related but different bacteria [7].

The global impact of SARS-CoV-2 has been profound, causing widespread illness and significant mortality worldwide. Although the acute pandemic phase has subsided, SARS-CoV-2 infection continues to affect a substantial portion of the population, often leading to health consequences and complications in some Patients [13]. For this reason, understanding the effects of the virus, particularly its impact on metabolism and subsequent biochemical markers, remains a critical and intriguing area of research. It is plausible that VOCs associated with SARS-CoV-2 infection differ from those produced under normal metabolic conditions, providing potential markers for early detection and monitoring of the disease. To interpret such complex molecular data, chemometrics techniques, such as Principal Component Analysis (PCA), are often applied to analyze VOCs data and can significantly enhance diagnostic accuracy by enabling the identification of infection-specific patterns. Previous studies have applied GC-MS to investigate the VOCs profiles of COVID-19 Patients, including analyses of breath and skin emissions [14,15].

While these studies typically relied on larger cohorts and pattern-recognition models, the present work contributes by adopting a high-resolution, untargeted GC-MS strategy, enabling detailed molecular annotation and the identification of candidate compounds with potential mechanistic and diagnostic relevance. The presence of potential biomarkers associated with SARS-CoV-2 infection may pave the way for their use in rapid diagnostic tools for early screening, characterized by high sensitivity and specificity. Our goal was not to replace current diagnostic methods, but to explore, with an untargeted approach, a complementary, rapid, non-invasive screening approach. In particular, the approach was conceived to provide a binary diagnostic indication (presence or absence of infection), rather than to characterize specific viral variants, which would require different analytical strategies and larger epidemiological cohorts. We applied fully automated headspace solid-phase microextraction (HS-SPME) coupled with gas chromatography (GC) and time-of-flight mass spectrometry (ToF-MS) [16] to analyze the volatilome of sebum and sweat samples collected from individuals with and without a diagnosis of SARS-CoV-2 (detected using standard diagnostic tests). Since previous studies have demonstrated that sweat and sebum are significant routes for the elimination of VOCs, making them ideal candidates for VOCs analysis [8,9], samples of this unique VOCs chemical fingerprint were collected non-invasively through gentle axillary swabbing using a cotton gauze. Such an approach could be particularly beneficial in contexts where rapid diagnostics are urgently required, such as remote locations or resource-limited healthcare systems. The ability to identify specific VOCs associated with infection would support future development of portable or wearable devices, such as electronic noses (e-noses) or smart patches, capable of on-site detection of disease biomarkers. Previous studies have demonstrated the feasibility of e-noses to detect VOCs patterns associated with diseases like lung cancer [17] and diabetes [18], reinforcing the potential of this method. Thus, precision medicine could increasingly rely on individual VOCs profiles, offering the possibility of more personalized diagnostic and therapeutic strategies.

## 2. Materials and Methods

The overall experimental workflow consisted of four main steps: 1. non-invasive collection of sweat and sebum samples; 2. storage and transport under controlled conditions; 3. HS-SPME-GC/ToF-MS analysis with automated sample handling; 4. multivariate chemometric analysis for feature selection and classification.

### 2.1. Study Design and Participant Selection

This study was designed as an observational, exploratory analysis to identify volatile biomarkers in sweat and sebum. The study protocol was approved (Protocol IRB 104/2024) by the Institutional Review Board (IRB) of the Department of Medicine, University of Udine (Italy). A total of 51 individuals participated in this study, including 27 Patients (Patients) with a recent diagnosis of SARS-CoV-2 infection and 24 control subjects (Controls). Patients were recruited consecutively: Participants were selected from those followed by the Division of Infectious Diseases, Azienda Sanitaria Universitaria Friuli Centrale (ASUFC), Udine, and Controls were enrolled at the Department of Medicine, University of Udine. No randomization or matching was applied, except for sex distribution (12 males and 15 females in the Patient group; 11 males and 13 females in the Control group). The control group was carefully selected to exclude individuals with any known respiratory infections, systemic inflammatory conditions, or recent acute illnesses, to enhance the reliability and specificity of the comparison.

Inclusion criteria included age ≥ 18 years (age distributions: patients were aged 77 ± 13 yrs. old, while controls were aged 53 ± 22 yrs. old), ability to provide informed consent, and, in the case of the patient group, a recent confirmed diagnosis of SARS-CoV-2. Exclusion criteria included severe COVID-19 requiring intensive care, presence of significant comorbidities (e.g., advanced cardiovascular or pulmonary diseases), dermatologic conditions affecting the sampling area, and active smoking within 7 days before sample collection.

All participants were free of skin diseases and received the same pre-sampling instructions (e.g., avoiding soaps, perfumes, or deodorants for 12 h). Written informed consent was obtained from all participants.

### 2.2. Diagnostic Criteria and Ethical Approval

Patients had a confirmed diagnosis of SARS-CoV-2 infection based on standard reverse transcription polymerase chain reaction (RT-PCR) on a nasopharyngeal swab. The diagnostic assay targeted the E gene (screening), and the RdRp and N genes (confirmation). A cycle threshold (Ct) value of ≤36 for at least one of the target genes was used to define a positive result. RT-PCR was performed using a LightMix^®^ Modular SARS and Wuhan CoV E-gene kit (TIB Molbiol Syntheselabor GmbH, Berlin, Germany) on a LightCycler^®^ 480 II instrument (Roche, Basel, Switzerland). While RT-PCR confirmed infection status, numerical Ct values and other laboratory parameters were not collected systematically, given the exploratory nature of the study. No interventional treatment was applied, but predefined inclusion/exclusion criteria were consistently enforced. For future validation studies, the integration of clinical metadata, including symptom profiles, comorbidities, and viral load metrics, will be essential to enhance reproducibility and diagnostic performance assessments.

Informed consent for the use of anonymized clinical data was obtained in accordance with the Helsinki Declaration. Hospital admission included routine inquiries about consent for the use of anonymized aggregate data for research purposes, facilitated by the General Electronic Consents (GECO) system. Relevant patient data were extracted by a team of physicians from the hospital’s electronic health record system (INSIEL, Trieste, Italy). Demographic, clinical, and diagnostic data were collected for all participants to ensure transparency and reproducibility.

### 2.3. Sampling and Analytical Procedure

Samples of sweat and sebum were collected by gently swabbing the skin in the axillary area using sterile cotton gauze. Participants were instructed to avoid soaps and deodorants for 12 h before sampling. Care was taken during the collection of the samples to avoid contamination by the environment, clothes, or other body areas.

Immediately after the swab, the gauze was placed in a sterile 10 mL polypropylene vial (Agilent Technologies Italia SpA, Cernusco sul Naviglio, Milano, Italy) with a pierceable cap, which was stored in a portable refrigerator at 4 °C and transported within a few hours to the laboratory of analysis (Faculty of Agricultural, Environmental, and Food Sciences, Free University of Bolzano). An anonymous code identified each vial. In the laboratory, the vials were stored at −80 °C until the analysis (within one month). Sample stability was validated during preliminary method setup. Analysts were blinded to sample origin (Patient or Control).

Volatile organic compounds (VOCs–volatilome) analysis was performed by solid-phase micro extraction (SPME) gas chromatography (GC) coupled with time-of-flight mass spectrometry (ToF-MS) (HS-SPME-GC/ToF-MS, LECO, St. Joseph, MI, USA) [16]. The SPME fiber used was a triphasic 1 cm 50/30 µm CARBOXEN/DVB/PDMS StableFlex™, needle size 23 ga, purchased from Merck Life Science SrL (Milan, Italy). Standard compounds (C4-C22 series of fatty acid ethyl esters in dichloromethane) used to calculate the retention index were purchased from Merck Life Science SpA (Milan, Italy). The vials were processed by analytical instrumentation using a pre-set method, without opening or other handling procedures. This ensured minimal sample degradation or contamination. The herein described SPME vials have a pierceable silicon septum, so no handling was necessary after their sealing.

The volatilome profile was characterized by HS-SPME-GC/ToF-MS on a LECO Flux BT 4D instrument (LECO, Mönchengladbach, Germany). The instrument was equipped with a PAL-II autosampler, and all described operations were performed automatically. For each analysis, the sample was pre-conditioned at 40 °C with 300 rpm stirring for 15 min. Then, the pre-conditioned SPME fiber was inserted into the vial, maintaining a 1 cm distance from the sample, and then the fiber in the vial was conditioned at 40 °C with 300 rpm stirring for 45 min. The fiber was then transferred to the 240 °C heated GC inlet and desorbed/injected from the heated inlet to the column by the He flow. The injection/desorption time was 6 min, and the GC inlet was set at 240 °C. In the gas chromatograph, the column used was a polar MEGA-WAX spirit column (PEG phase) 40 m/0.18 mm/0.30 μm (MEGA, Milan, Italy). The injection was done in splitless mode. The carrier gas (He) flow rate was 1 mL.min^−1^. The temperature ramp of the GC oven was 40 °C for 6 min (injection), then from 40 °C to 180 °C at 3 °C/min, then 180 °C to 240 °C at 10 °C/min, and 1 min at 240 °C (consequently, LRI attributed to compounds eluting above 180 °C might deviate considerably from their reference values). Detection was done with a pre-tuned time-of-flight (ToF) detector, according to the following parameters: acquisition rate = 5 spectra.s^−1^, acquisition mass range = *m*/*z* 35-650, extraction frequency = 32 kHz. The processing software ChromaToF^®^ (ver. 2021, LECO Corporation, Berlin, Germany) was used to process the GC-MS obtained automatically, providing identification and tentative assignments of compounds via spectral comparison with the NIST library 2017 (NIST MS search 2.3). Linear retention indexes were calculated against the even-carbon-containing ethyl ester of saturated linear fatty acids from C4 to C22. Samples were disposed after analysis.

Daily performance checks ensured analytical reliability: internal standards, blanks, retention time stability, and detector sensitivity were routinely verified. All materials were VOC-free certified. Procedural blanks and ambient air monitoring minimized background interference. These precautions align with recent recommendations on minimizing exogenous influences in volatilome analysis [15].

### 2.4. Data Processing and Statistical Analysis

The acquired data were furthermore exported as .mzData files and process on the MzMine2 platform (http://mzmine.github.io/features.html (accessed on 30 October 2025)), using version 2.53, for automated feature alignment.

On the aligned dataset (consisting of 8006 features), a PCA was performed (see next paragraph) to further filter the dataset. Namely, only the variables displaying the top 10% (in absolute value) positive and negative loadings from each of the first three Principal Components were retained for further analyses.

An empirical Bayes test (significance level = 5%) was also run, with Benjamini–Hochberg post hoc correction, and variable exclusion by *p*-value (only significant ones were retained). This set of variables was then applied to build a heat-map by hierarchical-clustering on observations and features, with an associated hierarchical tree plot (Figure S1).

The final dataset applied for Partial Least Squares Discriminant Analysis (PLS-DA) and empirical Bayes classification (see next paragraph) counted 3051 features out of the initial 8006. A targeted inspection of the results was also implemented.

The statistical analysis was performed using the XLSTAT add-on for Excel (Addinsoft, Paris, France). ANOVA (analysis of variance) and the Tukey HSD (honestly significant difference) post hoc tests were used to identify the variables significantly differentiating Controls from Patients. Principal Component Analysis was used as an exploratory tool, whereas Partial Least Squares Discriminant Analysis (PLS-DA) was used for multivariate discrimination between Controls and Patients. The best-obtained model was selected automatically by PRESS minimization, automatic variables centering and reduction, and Leave-One-Out cross-validation. For every run, 15 samples randomly selected from the dataset were applied in validation for PLS-DA.

## 3. Results

To uncover potential biomarkers for SARS-CoV-2, the volatile organic compounds (VOCs) profile from sweat and sebum samples was analyzed by HS-SPME-GC/ToF-MS. The samples were obtained from subjects who were instructed to avoid the use of soaps or deodorants for 12 h before sampling. This precaution was necessary to minimize external interferences and to ensure that the detected volatile organic compounds (VOCs) originated from endogenous metabolism rather than extrinsic contaminants. The goal was in fact to explore potential biomarkers associated with infection, which could potentially be implemented in a diagnostic test for SARS-CoV-2. Although the methodological approach was designed to maximize specificity in the volatilome characterization, in an applied diagnostic setting, sample collection protocols could certainly be adapted to be more user-friendly while preserving analytical accuracy, once the specific biomarkers are identified.

**Figure 1:**
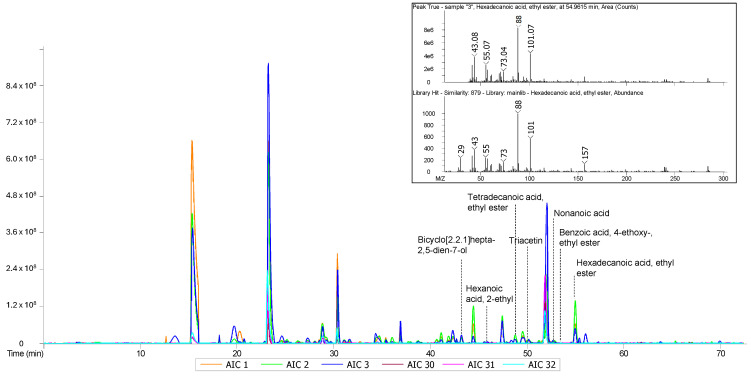
Selected GC chromatograms of the analyzed samples (raw data). Samples 1, 2, and 3 are for known negative cases (Controls). Samples 30, 31, and 32 are for known positive cases (Patients). Examples for compounds of interest (discussed below) have been indicated in the chromatogram. Inset: EI (70 eV)/MS spectrum and relative library hit (NIST 2027) for ethyl hexadecanoate.

The volatilome resulting from the HS-SPME-GC/ToF-MS analysis of sweat and sebum is reported in the chromatographic traces of Figure 1.

**Figure 2:**
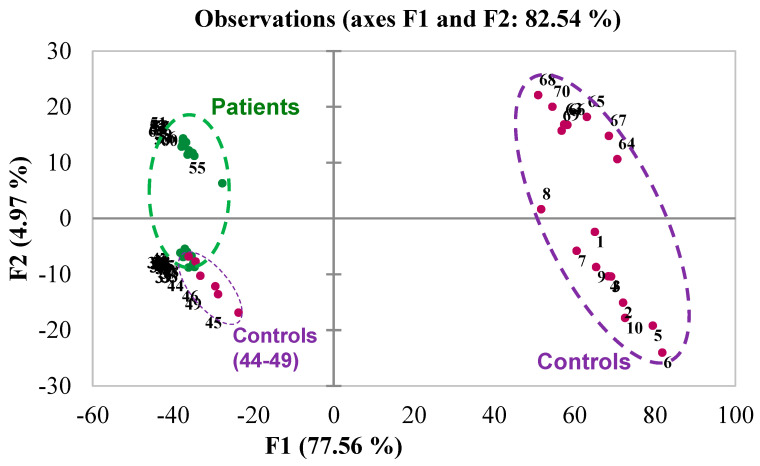
Scores plot of the PCA model (PC1 vs. PC2) built on the refined dataset (GC-MS, 3051 variables). Samples (observations) are indicated by numbers. Green labels = PATIENTS, Purple labels = CONTROLS. Ellipses are included just to help interpretation, and bear no statistical significance. Labels: sample numbers.

The acquired data on 51 samples (27 Patients and 24 Controls) were pre-processed using the software ChromaToF (LECO). In summary, the samples were automatically processed by chromatographic deconvolution of the present spectral features. The obtained initial peak lists were then refined from artifacts and background removal. As part of the dataset pre-processing, molecular species with too sparse distributions or anomalous effects (outliers) among the samples were removed. We obtained a refined dataset that was applied for an explorative multivariate analysis by PCA. The results of this elaboration are presented in Figure 2.

The PCA model could not effectively separate the two groups entirely. Samples 44–49 were Controls and resulted in being associated with Patients in the PC1 vs. PC2 projection. To understand if this dataset could be useful for building a predictive model for discriminating Patients from Controls, a Partial Least Squares Discriminant Analysis model was built on the PCA-filtered dataset (3051 features, as reported in Section 2).

In addition, an empirical Bayes test was implemented on the observations, in order to extract significantly discriminating features (Figure S1). The results are presented as a heat-map, adding also a hierarchical clustering for observations and features, showing their associations (Supporting Information, PATIENTS: samples 30–43, 47, 51–62; CONTROLS: samples: 1–10, 44–46, 48–50, 63–70). Overall, the heatmap distinguished neatly three main clusters, in which samples 51–62 (PATIENTS) and 1–10 + 63–70 (CONTROLS) were far apart. The remaining samples were clustered in between.

The PLS-DA results are reported in Table 1. Fifteen randomly selected samples were applied in every run as a validation dataset. For the computation of the model, prior probabilities were not considered. In addition, no equality of the within-class covariance matrices was imposed for the calculation. A PLS-DA with 2 components was obtained by imposing predicted residual error sum of squares (PRESS) minimization. The resulting model (2-factor model) was characterized by very good quality parameters, such as Q2-cum. = 0.727, R2Y-cum. = 0.820, and R2X-cum = 0.809. The PLS-DA results confirmed the preliminary exploratory observations highlighted by PCA, as a small percentage of false positives (control samples incorrectly assigned as cases) were again observed. Notably, the model demonstrated consistent statistical performance, achieving zero false negatives, meaning that every infected patient was correctly identified. A false-positive rate of up to 28.6% was observed among control samples during validation. This result is consistent with the exploratory design of the study, which did not include stratification by lifestyle, physiological, or environmental variables that can influence baseline VOC composition in healthy individuals. Importantly, the absence of false negatives supports the robustness of the discriminative signal, although model specificity will require further refinement and confirmation in larger, independent cohorts.

In Table 1, the variables’ importance in projection (VIP index, model with 2 factors) for the first ten most important variables are reported. The variables are ranked by VIP, and a tentative assignment is provided. All the reported species (tentatively assigned) presented higher values for the Controls.

**Figure 3:**
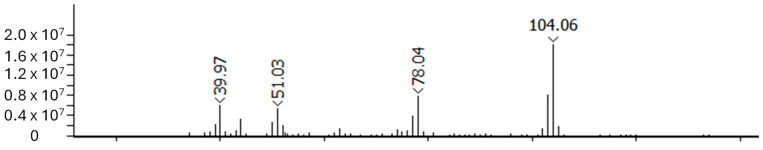
Recorded ToF-MS spectrum (EI = 70 eV) tentatively assigned to 2-methylbenzenemethanol acetate (examples of spectra from sample 34). Assignment given upon comparison with NIST 2017 spectral library.

Therefore, a targeted inspection of the VIPs list allowed us to tentatively identify a potential marker for the infection. 2-methylbenzenemethanol acetate (R.t. = 18.2 min, base peak = *m*/*z* 104.1) was tentatively identified (after validation by ANOVA) to be significantly higher in the Patients (height of the peak applied as dependent variable with LOG(10) correction, F-val. = 9.701, *p*-val. = 0.003, α = 95%). The experimentally recorded MS spectrum for this feature is reported in Figure 3.

## 4. Discussion

In clinical diagnostics, the identification of rapid, accurate, and non-invasive biomarkers is essential to tackle the challenges posed by emerging infectious diseases, such as SARS-CoV-2. While current methods like RT-PCR testing are reference standards because they are highly specific, their invasiveness, reagent dependency, and diagnostic sensitivity limitations highlight the need for alternative approaches. Innovative approaches based on the analysis of the volatilome naturally emitted by the body can be a promising method to detect infections at an early stage and manage them.

In this context, the present study was conceived to detect a general infection-related signature, providing a binary diagnostic output (presence/absence of infection), rather than distinguishing between viral variants, an endeavour requiring different analytical strategies and larger cohorts. RT-PCR analysis of nasopharyngeal swabs was used as the reference method to confirm SARS-CoV-2 status in participants, ensuring alignment with the clinical gold standard. The VOC-based discrimination was therefore benchmarked against RT-PCR-confirmed infection status. The aim here was to assess whether untargeted GC-MS can identify infection-associated molecular features, not to benchmark diagnostic performance against RT-PCR.

Chemometrics is the branch of analytical chemistry focused on extracting relevant information from complex datasets and can play a pivotal role in processing the huge datasets derived from VOC analysis, enabling robust discrimination between pathological and healthy states. Although the number of Patients included in this study was relatively small, it was appropriate for the analytical aim, which focused on identifying discriminant molecular markers using a high-precision, non-targeted GC-MS approach. The methodology enabled detailed spectral analysis and statistically sound multivariate modelling, supporting future validation studies on larger, more diverse populations.

Sweat and sebum samples were collected from subjects diagnosed with (Patients) and without (Controls) a confirmed SARS-CoV-2 infection, verified through RT-PCR analysis of nasopharyngeal swabs. Coupled with highly automated HS-SPME-GC/ToF-MS analysis, this non-invasive sampling ensured minimal inconveniences and preserved sample integrity. This approach provides a reliable and participant-friendly platform for volatilome studies. The protocol for gas chromatography (GC) and mass spectrometry (MS) analysis was outlined by Sinclair et al. [8,9], and Darnal et al. [16]. The data obtained from the analysis were preliminarily processed with Principal Component Analysis (PCA) to explore and select the most significant features from the data (Figure 2). The Loadings from PCA were then used to filter the dataset, to remove less relevant variables for discrimination. Subsequently, Partial Least Squares Discriminant Analysis (PLS-DA) was applied using the refined set of chemical variables (the VOCs).

With this approach, it was possible to discriminate between Patients and Controls based on the VOC patterns. Initially, 20–30% of the Control samples were misclassified as Patients, indicating a notable false positive rate. To improve accuracy, further variable selection was applied, refining the model by removing interfering signals. This led to improved classification performance. Cross-validation and application to randomized validation sets demonstrated >95% accuracy overall. Specifically, Controls were correctly identified in 88.24% of cross-validation cases and 71.43% in the validation datasets (Table 2).

Patients were correctly assigned 100% of the time in both analyses. These figures should be interpreted as internal cross-validation results rather than as diagnostic accuracy estimates, because the present study was designed as a proof-of-concept for candidate biomarker discovery, not for diagnostic validation. In this framework, the cohort size (n = 51) was appropriate for untargeted high-resolution GC/ToF-MS, where each sample yields thousands of variables, and the analytical focus is on detecting discriminant signals rather than estimating clinical performance. Accordingly, the reported >95% value reflects internal model consistency and does not imply generalizability or real-world diagnostic performance, which will require external validation on larger, independent cohorts.

The chemometric model allowed us to achieve robust classification, correctly identifying all positive samples within the dataset. A limited number of false positives were observed among Controls. The high classification accuracy observed in cross-validation does not equate to a full diagnostic validation. Indeed, performance metrics such as sensitivity, specificity, ROC curves, or confusion matrices were not computed here, as the limited cohort size and the absence of an external test set preclude reliable generalization. Accordingly, this study focused on the identification of discriminant VOC features rather than on diagnostic accuracy estimation. The design is consistent with a biomarker-discovery scope. The cohort size (n = 51) was adequate for untargeted GC/ToF-MS analysis, which prioritizes analytical resolution per sample over statistical generalization. Despite this, the PLS-DA model achieved robust internal performance (Q^2^_cum = 0.727, R^2^Y = 0.820) with no false negatives, supporting the reproducibility of the discriminative signal within this proof-of-concept framework. Nonetheless, it is clear that broader external validation will be required to confirm biomarker stability across populations differing in age, comorbidities, and environmental exposures. This is the focus of ongoing work, which will establish the generalizability and clinical reliability of the proposed biomarkers.

Given the use of high-resolution, untargeted GC-MS, the chosen sample size was appropriate for identifying molecular markers with mechanistic significance, offering greater analytical depth than large-scale sensor-based studies [14]. Although classical multivariate techniques such as PCA and PLS-DA were employed, alternative or other approaches, including univariate tests (e.g., Mann–Whitney U), ROC analysis, and machine learning algorithms (e.g., neural networks), could provide further insights during future validations. These methods, however, require larger datasets and were beyond the scope of this initial profiling study. The integration of these methods will be essential to advance from exploratory chemometric modeling toward clinically validated diagnostic tools. PCA and PLS-DA were employed to explore data structure and identify discriminant signals, not as clinical diagnostic models per se. However, these models can be translated into automated diagnostic tools through embedded algorithms in sensor-based platforms, ultimately delivering simplified, clinically actionable outputs. This integration would enable the application of VOC-based analysis in clinical workflows in point-of-care or hospital settings without requiring specialized knowledge of multivariate statistics.

The main objective of this work was to explore potential biomarkers associated with SARS-CoV-2 infection by comparing healthy individuals and confirmed COVID-19 Patients. Rather than replacing current diagnostic methods, this approach aims to offer complementary, non-invasive testing tools. In the context of precision medicine, the characterization of the volatilome offers a diagnostic approach tailored to the specific metabolic and microbial profiles of the subjects. It addresses the inter-individual variability and the environmental influences and could improve diagnostic precision and reduce the risk of misclassification.

The promising cross-validation accuracy supports the possible translational relevance of the refined model. Given the 70–80% sensitivity of current antigenic and molecular tests [19,20], non-invasive tools like skin swabs could provide meaningful advantages. Nonetheless, it is necessary to highlight that the impact of soaps, perfumes, deodorants, or other cosmetic products on the volatilome must be carefully considered, as these may alter VOC profiles. Further studies should aim to identify markers unaffected by such confounders, or to isolate the interfering signal from cosmetics. However, although potential confounding factors such as diet, medication, and personal hygiene products were not controlled beyond the 12-h restriction on soaps and deodorants, this choice reflects the exploratory and deliberately realistic design of the study. The inclusion of participants without lifestyle stratification inherently increased within-group variability, thereby testing the robustness of infection-related VOC signatures under real-world conditions. The persistence of significant discriminatory features despite this variability supports their potential biological relevance.

The specific chromatographic features identified in this study were obtained through a fully untargeted approach. No pre-treatment of samples was applied, and no assumptions were made regarding the molecular classes involved. Further research is required to confirm and characterize the compounds responsible for distinguishing between positive and negative cases in a larger and more diverse population. For instance, this includes understanding how the volatilome may behave in the presence of other pathological or environmental factors, to establish the robustness of potential diagnostic markers. Preliminary data analysis tentatively identified a key compound of interest (2-methylbenzenemethanol acetate), which was reported as a promising marker for infection. Its identification was given based on GC-MS data and comparisons with the NIST spectral library. The metabolism of compounds similar to 2-methylbenzenemethanol acetate can occur through various biochemical pathways in humans, particularly involving aromatic derivatives and microbial interactions [21]. While direct evidence for 2-methylbenzenemethanol acetate’s metabolism is lacking, investigations already done on related compounds can give an idea of how the body can process similar substances. It is important to consider that VOCs can originate not only from host metabolism but also from microbial activity on host skin. In fact, recent studies have demonstrated that certain VOCs, including esters and alcohols, can be produced by bacteria such as *Xenorhabdus indica* strain AB [22] and fungi like *Aspergillus flavus* [23]. While our untargeted GC-MS approach allowed the identification of 2-methylbenzenemethanol acetate as a discriminant compound in COVID-19 Patients, we cannot exclude that its presence may be influenced by microbial populations inhabiting the axillary region. 2-Methylbenzenemethanol acetate, the most discriminant feature identified in this study, is an aromatic ester that may arise from the acetylation of 2-methylbenzyl alcohol, a compound linked to amino acid and/or microbial metabolism. Aromatic esters are known to form through acetyl-CoA–dependent acetyltransferase activity acting on aromatic alcohols, a mechanism widely reported in microbial and yeast systems [24,25]. To the best of our knowledge, a direct biosynthetic route for 2-methylbenzenemethanol acetate in humans or microbiota has not yet been described. Thus, the following remains a plausible hypothesis.

Viral infections such as SARS-CoV-2 are known to induce oxidative stress, inflammatory responses, and alterations in the skin and mucosal microbiome [26,27], all of which can influence ester-forming enzymatic activity. It is therefore plausible that the observed increase in this compound in infected subjects reflects host–microbiome metabolic interactions modulated by the infection rather than a direct viral product. The absence of this compound in analytical blanks and its significant enrichment in the patient group (*p* = 0.003, VIP > 1) support a biological, rather than environmental, origin. Further investigations combining targeted metabolomic and metagenomic approaches will be required to elucidate its metabolic pathway and confirm its diagnostic specificity in independent cohorts. Nevertheless, it is worth noting that the consistent increase in this compound is observed only in SARS-CoV-2-positive individuals, suggesting a potential link between viral infection and the modulation of host–microbiome interactions. The infection might either directly or indirectly alter the skin microenvironment or immune regulation, favoring microbial proliferation or metabolic shifts that lead to the production of this compound. Nonetheless, the absence of this compound in the control group reduces the likelihood of a widespread fungal/microbial source, although further studies integrating mycobiome profiling would be needed to fully clarify this possibility. Moreover, the use of volatile organic compounds (VOCs) in exhaled breath has emerged as a promising non-invasive diagnostic approach for SARS-CoV-2 infection. A recent study [28] identified VOCs biomarkers that could distinguish between COVID-19 cases and non-COVID-19 illnesses during the circulation of the Delta variant. However, the emergence of the Omicron variant significantly altered the volatilome profile, showing that there was a need to identify a different set of VOCs to maintain diagnostic accuracy.

In the current study, 2-methylbenzenemethanol acetate is reported in association to SARS-CoV-2 infection. This compound highlights its possible role as a diagnostic indicator, reflecting shifts in metabolic processes induced by the virus. While this evidence is promising, further studies are necessary to validate its specificity and sensitivity compared to other candidate VOCs. Nonetheless, these findings provide a foundation for follow-up investigations to explore its diagnostic utility and specificity. Moreover, investigating the response of the volatilome to confounding factors such as cosmetics or environmental exposure will help refine the applicability of volatile biomarkers in diagnostic settings. By deepening our understanding of the biochemical and environmental influences on these VOCs, it is possible to unlock their potential as reliable biomarkers for infectious and inflammatory diseases.

Further research should also investigate the biological mechanisms underlying infection-associated volatilome changes, as detected here with headspace GC-MS. Clarifying these metabolic pathways could support the development of precision medicine strategies and improve the design of effective diagnostic and therapeutic tools. This information is valuable for the development of specific rapid sensors. Refining sensor technology, potentially using nanotechnology or microfluidics, could further improve sensitivity and analytical efficiency in detecting infection-related VOCs, including those associated with SARS-CoV-2.

## 5. Conclusions

VOCs detection from sweat or sebum using HS-SPME-GC/ToF-MS offers a non-invasive and accessible alternative to conventional diagnostic methods for the assessment of the SARS-CoV-2 infection. A key finding was the identification of 2-methylbenzenemethanol acetate, which emerged as a candidate marker associated with infection and inflammation. However, although strongly associated with SARS-CoV-2, its presence may not be exclusive to this infection, and further studies are needed to investigate its disease specificity. If fully automated, the protocol could be implemented as a point-of-care solution (e.g., in emergency departments). In a pandemic context, this methodology could help mitigate the shortage of molecular reagents and consumables. Moreover, this approach requires minimal consumables aside from the equipment itself, making it cost-effective compared to RT-PCR, especially in large-scale applications. This may reduce per-patient testing costs and enhance sustainability in both routine and emergency diagnostic scenarios.

VOCs detection facilitates early infection detection, including in asymptomatic individuals, while minimizing patient discomfort and improving compliance. However, further validation is needed to confirm its specificity and reliability in clinical contexts, and its broader applicability to infections beyond respiratory illnesses should be explored.

Combining advanced sensors with artificial intelligence (AI) may transform diagnostic practices. If validated, specific VOCs may serve as molecular targets for future sensor development. By using AI, the data collected from VOCs detected by sensors might be elaborated more accurately and support the creation of personalized, efficient diagnostic systems capable of rapid, on-site detection of infections. Building on these findings, future development will require validation in larger, diverse cohort biomarker specificity studies across different conditions. As technology advances, particularly through breakthroughs in nanotechnology and machine learning, this integrated approach could develop into a clinically applicable tool, combining sensitivity, convenience, and early detection capability. Notably, several studies have already demonstrated the applicability of e-nose technology for detecting SARS-CoV-2 infection, using pattern-recognition approaches with promising sensitivity and specificity [29,30,31,32]. While our study did not aim to replicate or benchmark against these sensor-based methods, it focused instead on identifying specific molecular VOCs using untargeted GC-MS analysis. This mechanistic approach may pave the way to targeted sensor arrays, providing a complementary foundation to current pattern-based technologies.

## Figures and Tables

**Table 1:** PLS-DA model: First ten variables by importance in projection (VIP index, model with 2 factors).

Variable Label	VIP (2-Factors Model)	*m*/*z ^a^*	Average R.t./Min–Experimental LRI	Tentative Assignments
x.7050	2.28	40.0	55.1–2361	Ethyl hexadecanoate
x.6423	2.09	121.1	52.4–2094	Ethyl 4-ethoxybenzoate
x.972	2.09	79.0	15.7–1174	Not assigned
x.4854	2.03	41.1	42.8–1802	Not assigned
x.4906	1.90	44.0	43.2–1815	Bicyclo [2.2.1] hepta-2,5-dien-7-ol
x.5961	1.82	60.0	50.0–2116	Triacetin
x.5289	1.70	40.0	45.6–1912	2-Ethylhexanoic acid
x.6506	1.62	40.0	52.7–2176	Nonanoic acid
x.6457	1.58	147.0	52.6–2248	Not assigned
x.5806	1.53	40.0	48.9–2061	Tetradecanoic acid, ethyl ester

*^a^* The mass shown does not reflect the base peak of the spectrum, but that among all that was assigned the highest VIP. LRI, Linear Retention Index.

**Table 2:** Results of the partial least squares discriminant analysis modelling (PLS-DA) of the evaluated samples. (**A**) Training set-confusion matrix, and (**B**) validation set-confusion matrix.

(**A**) Confusion matrix for the training dataset				
**from\to**	**PATIENT**	**CONTROL**	**Total**	**% correct**
PATIENT	19	0	19	100.00%
CONTROL	2	15	17	88.24%
Total	21	15	36	94.44%
(**B**) Confusion matrix for the validation dataset (15 randomly selected samples)				
**from\to**	**PATIENT**	**CONTROL**	**Total**	**% correct**
PATIENT	8	0	8	100.00%
CONTROL	2	5	7	71.43%
Total	10	5	15	86.67%

## Data Availability

The data presented in this study are available on request from the corresponding author. The data are not publicly available due to privacy.

## Supplementary Materials

The following supporting information can be downloaded at: https://www.mdpi.com/article/10.3390/metabo16030158/s1, Figure S1: Heatmap of empirical Bayes–selected variables (α = 0.05, Benjamini–Hochberg), with hierarchical clustering of samples and features.

## Abbreviations

The following abbreviations are used in this manuscript:

BT	BenchTop
EI	Electron Ionisation
ERDF	European Regional Development Fund (FESR)
GC	Gas Chromatography
HS	Headspace
HS-SPME	Headspace Solid-Phase Microextraction
IRB	Institutional Review Board
LRI	Linear Retention Index
MS	Mass Spectrometry
NIST	National Institute of Standards and Technology
PCA	Principal Component Analysis
PLS-DA	Partial Least Squares Discriminant Analysis
PRIN	Progetti di Rilevante Interesse Nazionale
RT-PCR	Reverse Transcription Polymerase Chain Reaction
SPME	Solid-Phase Microextraction
ToF-MS	Time-of-Flight Mass Spectrometry
VOC/VOCs	Volatile Organic Compounds
