# Supplementary material for: Untargeted Sweat and Sebum Volatilomics by HS-SPME-GC/ToF-MS for the Identification of SARS-CoV-2-Associated Biomarkers
Source: Metabolites. 2026 Feb 27;16(3):158. doi: 10.3390/metabo16030158 (PMC13028495; doi:10.3390/metabo16030158)
Supplement: Supplementary file 1 [file metabolites-16-00158-s001.zip › metabolites-4116808-supplementary.pdf]

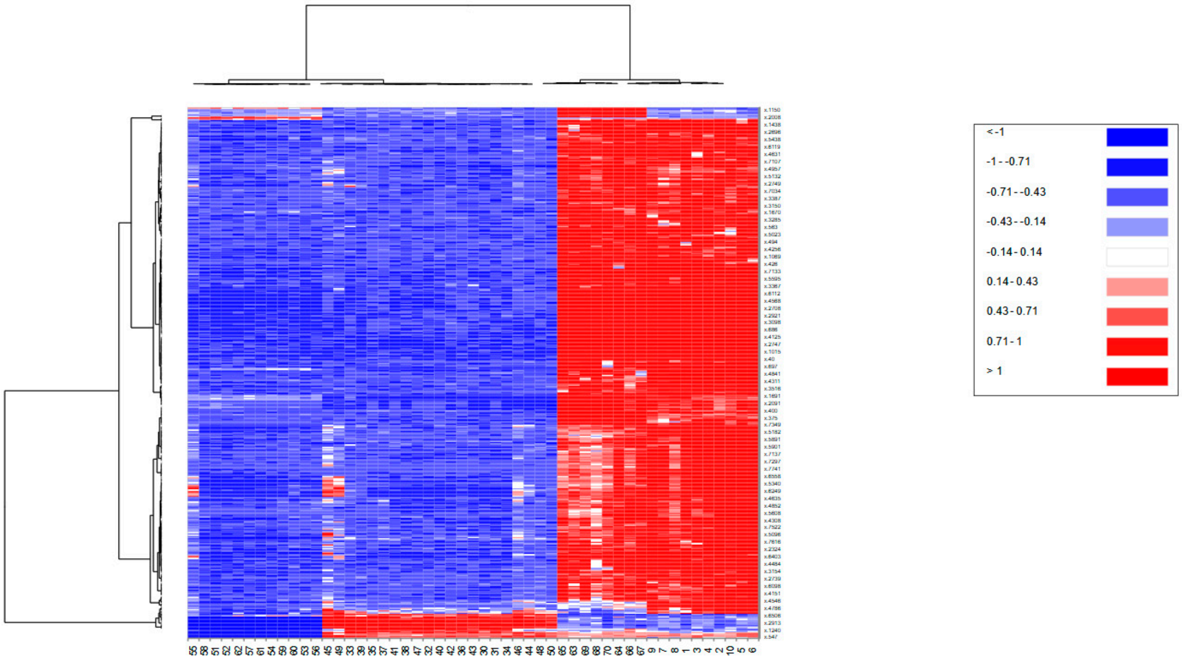

Figure S1. Heatmap of empirical Bayes–selected variables (α = 0.05, Benjamini–Hochberg), with hierarchical clustering of samples and features.
